# Incidence trends and survival prediction of hepatoblastoma in children: a population-based study

**DOI:** 10.1186/s40880-019-0411-7

**Published:** 2019-10-24

**Authors:** Jincheng Feng, Georgios Polychronidis, Ulrike Heger, Giovanni Frongia, Arianeb Mehrabi, Katrin Hoffmann

**Affiliations:** 1 Department of General, Visceral and Transplant Surgery, University of Heidelberg, Im Neuenheimer Feld 110, 69120 Heidelberg, Germany; 2 Department of Pediatric Surgery, University of Heidelberg, Im Neuenheimer Feld 110, 69120 Heidelberg, Germany

**Keywords:** Hepatoblastoma, Liver surgery, Liver transplantation, Pediatric surgery, SEER, Incidence, Overall survival, Nomogram

## Abstract

**Background:**

Hepatoblastoma is a rare disease that nevertheless accounts for the majority of liver malignancies in children. Due to limited epidemiological data, therapy for hepatoblastoma tends to be individualized. This study aimed to evaluate incidence trends of hepatoblastoma and to develop a nomogram to predict the survival of children with newly diagnosed hepatoblastoma on a population-based level.

**Methods:**

Individuals up to 18 years of age with hepatoblastoma recorded in 18 registries of the Surveillance, Epidemiology, and End Results (SEER) database between 2004 and 2015 were examined. Joinpoint regression analyses were applied to assess incidence trends in annual percentage change (APC). Multivariable Cox regression was used to identify factors associated with overall survival (OS). A nomogram was constructed to predict OS in individual cases based on independent predictors. Concordance index (C-index) and calibration curves were used to evaluate predictive performance.

**Results:**

Between 2004 and 2015, hepatoblastoma incidence increased significantly (APC, 2.2%; 95% confidence interval [CI] 0.5% to 3.8%, *P* < 0.05). In particular, this increase was observed among 2- to 4-year-old patients, males, and African–Americans. The 5- and 10-year OS rates were 81.5% and 81.0%, respectively. Age of 2 to 4 years, African–American ethnicity, and no surgery were independent predictors for short OS. Distant disease at presentation was found not to be an independent factor of survival. The nomogram had a C-index of 0.79 (95% CI 0.74–0.84) with appropriate calibration curve fitting.

**Conclusions:**

We constructed a nomogram that integrates common factors associated with survival for hepatoblastoma patients. It provides accurate prognostic prediction for children with hepatoblastoma.

## Background

Liver tumors among children are relatively rare, accounting for approximately 1% of all childhood malignancies [1–4]. The overall age-standardized rate of incidence (world standard) of liver tumors in children was 2.3 per million person-years between 2001 and 2010 worldwide [5]. Hepatoblastoma is the most common malignant liver tumor in children, with an increase in incidence of 4.3% per year, from 1992 to 2004, in children younger than 19 years [1, 4, 6, 7]. Hepatoblastoma is most frequently diagnosed in children less than 5 years of age [3, 7–9]. Treatment of hepatoblastoma includes chemotherapy, surgical resection, and liver transplantation [10]. Complete surgical resection is the first-line treatment for resectable hepatoblastoma at initial diagnosis, and liver transplantation is one of the main treatments for unresectable hepatoblastoma [11–13]. Survival outcomes have greatly improved over the past four decades [10, 11, 14, 15]. Although the prognostic factors and prognosis of hepatoblastoma have been reported in some population-based studies [8, 14, 16, 17], nomograms to predict long-term survival for patients with hepatoblastoma are still lacking.

Nomograms have been developed for some cancers and are accepted as reliable tools for individualized risk prediction [18–21]. Given the rarity of hepatoblastoma as well as the small sample sizes of retrospective studies [11, 15, 22–24], a large population-based study is needed. Therefore, we analyzed the incidence trends and clinical outcomes of hepatoblastoma in children using the data from 18 cancer registries of the Surveillance, Epidemiology, and End Results (SEER) database, which is supported by the Surveillance Research Program (SRP) of Division of Cancer Control and Population Sciences (DCCPS) of the National Cancer Institute (NCI), and constructed a prognostic nomogram to predict long-term survival of patients with hepatoblastoma.

## Methods

### Case selection from the SEER database

Data from the SEER database for year 2004–2015 were analyzed. The selection criteria were patients of 0 to 18 years old who were newly diagnosed with hepatoblastoma between January 1, 2004 and December 31, 2015. Exclusion criteria were unknown ethnicity, unknown surgical intervention, and lack of follow-up. The following variables were obtained from the SEER database for further analysis: sex, age at diagnosis, ethnicity, year of diagnosis, tumor size, tumor stage, alpha-fetoprotein (AFP) status, and surgery at the primary site.

### Definition of variables

Because the Union for International Cancer Control (UICC) tumor-node-metastasis (TNM) staging system is not used for hepatoblastoma cases in the SEER database, the SEER summary staging guidelines [25] were used to evaluate tumor stage in this analysis. The SEER variable “Summary Stage 2000 (1998+)” describes a surgical procedure that removes and/or destroys the tissue at the primary site performed as part of the initial work-up or first course of therapy. This enabled classifying cases into four categories: local, regional, distant, and unstaged. The SEER surgery codes recorded data on liver-directed surgical treatments, which were categorized as no surgery, liver resection, and liver transplantation. Sex was classified as male and female. The SEER program classified ethnicity into three categories: Caucasian ethnicity, African–American ethnicity, and other ethnicities (American Indian/Alaska Native, Asian/Pacific Islander). Age at diagnosis was classified into three groups: 0–1, 2–4, and 5–18 years old. Year of diagnosis was divided into three time periods: 2004–2007, 2008–2011, and 2012–2015. Tumor size was also classified into three categories: ≤ 5 cm, > 5 cm, and unknown. AFP status was classified into three categories: positive, negative, and unknown (according to the SEER database). Because all patient information in the SEER database is de-identified, this study was exempted from review by the Institutional Review Board.

### Statistical analysis

The age-standardized incidence of hepatoblastoma according to the 2000 US Standard Population was identified in the cancer registries of the SEER database [26]. The annual percentage change (APC) of incidence was calculated using the weighted least squares method. We analyzed the incidence of hepatoblastoma stratified by sex, age at diagnosis, and ethnicity. Joinpoint regression analyses (v. 4.6.0, IMS, Calverton, MD, USA) were used to analyze incidence trends from 2000 to 2015. Overall survival (OS) was estimated using the Kaplan–Meier method, and the log-rank test was used to compare survival curves. OS was defined as the period from diagnosis to death of any cause. For individuals who were still alive at the time of the last follow-up, OS was censored at the date of last follow-up or December 31, 2015, whichever came first.

A Cox proportional hazards model was applied for multivariate survival analysis. Those variables with a *P* value less than 0.05 in the univariate analysis were entered into a Cox proportional hazards model to identify independent predictors of OS. A nomogram was constructed based on the results of the multivariate analysis. Nomogram performance was measured using the concordance index (C-index). The larger the C-index, the more accurate the prognostic prediction proved to be. The nomogram was subjected to 1000 bootstraps resamples for internal validation. Calibration of the nomogram for 5- and 10-year OS was performed by comparing predicted survival with observed survival. The data were analyzed using SPSS version 20.0 for Windows (SPSS Inc., Chicago, IL, USA) and R software version 3.5.0 (https://www.r-project.org/). All *P* values were two-sided, and a *P* value < 0.05 was regarded as statistically significant.

## Results

### Patient demographics

Using the latest data released in April 2018, we identified 523 eligible individuals using the topography and morphology codes from the third edition of the International Classification of Diseases for Oncology (ICD-O-3). Of these 523 cases, we excluded 12 due to lack of data on ethnicity (*n* = 7), surgery (*n* = 2), or follow-up (*n* = 3). The remaining 511 cases were selected for this study. The median age at diagnosis was 1 year old (range 0–17 years). The majority of patients were males (62.6%), of Caucasian ethnicity (76.7%), and had a positive AFP status (82.4%). Surgery was performed on 425 (83.2%) patients. Of them, 341 (66.7%) received a liver resection, and 84 (16.4%) had liver transplantation. Baseline patient demographics and tumor characteristics are shown in Table 1.

**Table 1:** Demographic and clinicopathologic characteristics, and overall survival of patients with hepatoblastoma

Characteristic	Patients [cases (%)]	Deaths [cases (%)]	Overall survival rate (%)		*P* value
			5-year	10-year	
All patients	511	82	81.5	81.0	
Sex					0.139
Male	320 (62.6)	58 (70.7)	79.3	78.4	
Female	191 (37.4)	24 (29.3)	85.6	85.6	
Ethnicity					0.001
Caucasian	392 (76.7)	55 (67.1)	83.9	83.9	
African–American	44 (8.6)	15 (18.3)	58.4	58.4	
Others	75 (14.7)	12 (14.6)	82.5	79.0	
Age at diagnosis					0.013
0–1	323 (63.2)	41 (50.0)	85.8	85.8	
2–4	152 (29.7)	36 (43.9)	72.9	71.0	
5–18	36 (7.1)	5 (6.1)	79.9	79.9	
Year of diagnosis					0.108
2004–2007	150 (29.4)	35 (42.7)	76.6	75.8	
2008–2011	175 (34.2)	25 (30.5)	84.8	NR	
2012–2015	186 (36.4)	22 (26.8)	NR	NR	
Tumor size					< 0.001
≤ 5 cm	60 (11.7)	2 (2.4)	96.4	96.4	
> 5 cm	400 (78.3)	62 (75.6)	82.2	81.5	
Unknown	51 (10.0)	18 (22.0)	60.4	60.4	
Tumor stage					< 0.001
Local	231 (45.2)	17 (20.7)	91.0	91.0	
Regional	157 (30.7)	27 (32.9)	80.4	80.4	
Distant	109 (21.3)	34 (41.5)	65.7	65.7	
Unstaged	14 (2.7)	4 (4.9)	77.4	64.5	
AFP status					0.028
Negative	10 (2.0)	1 (1.2)	89.4	89.4	
Positive	421 (82.4)	61 (74.4)	83.4	82.7	
Unknown	80 (15.7)	20 (24.4)	70.9	70.9	
Surgery type					< 0.001
No surgery	86 (16.8)	46 (56.1)	43.2	39.4	
Liver resection	341 (66.7)	29 (35.4)	89.3	89.3	
Liver transplantation	84 (16.4)	7 (8.5)	90.1	90.1	

*NR* not reached, *AFP* alpha-fetoprotein

### Incidence trends

**Fig. 1:**
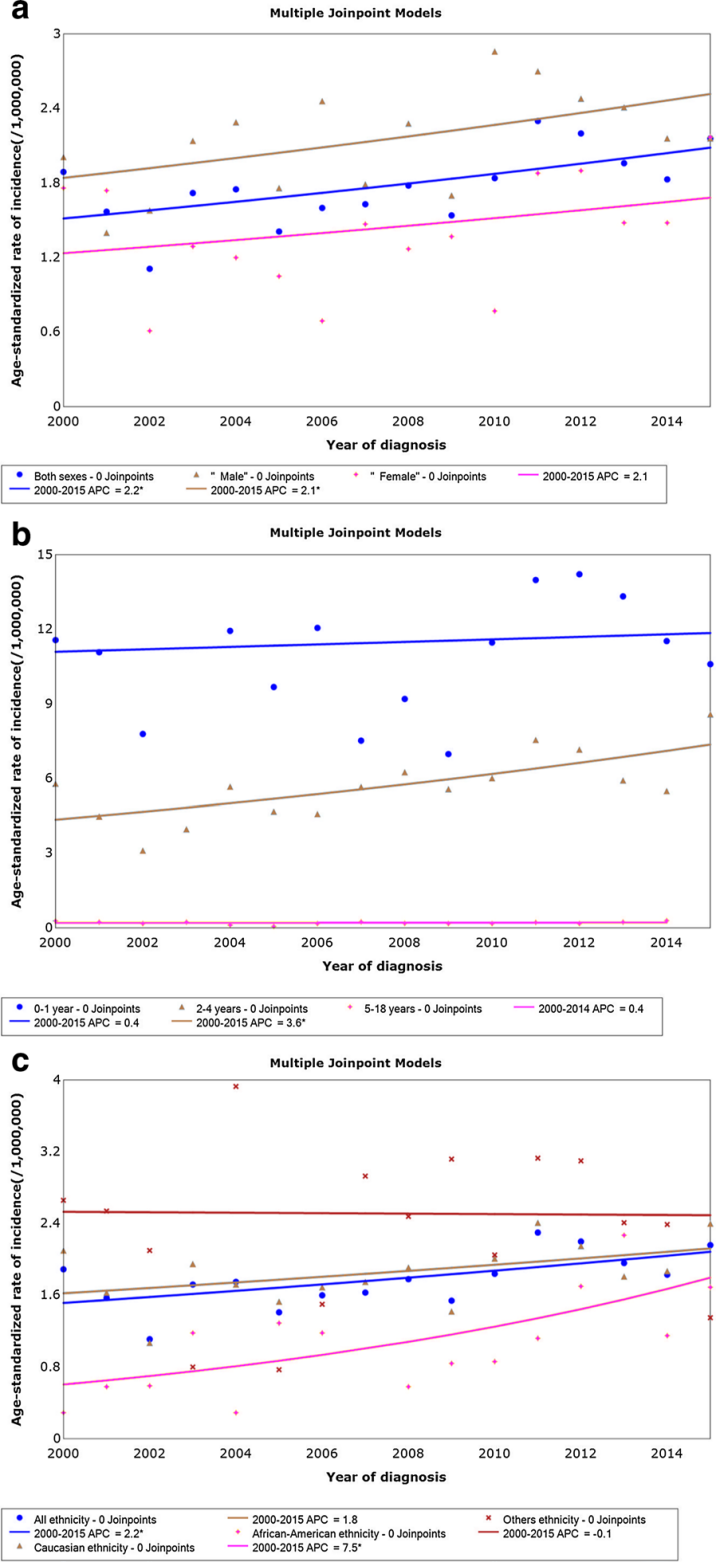
Hepatoblastoma incidence trends by sex (**a**), age at diagnosis (**b**), and ethnicity (**c**) among patients aged less than 18 recorded in 18 registries of the SEER database, 2004 to 2015. *APCs significantly different than zero at the 5% level, calculated based on a two-sided *t* test. *SEER* the Surveillance, Epidemiology, and End Results database, *APC* annual percentage change

The incidence of hepatoblastoma increased from 1.89 per 1,000,000 in 2000 to 2.16 per 1,000,000 in 2015, with an APC of 2.2% (95% confidence interval [CI] 0.5% to 3.8%, *P *= 0.014). We identified a significant increase in hepatoblastoma incidence from 2000 to 2015 in the male population with an APC of 2.1% (95% CI 0.1% to 4.2%, *P* = 0.047). The rise in incidence was not significant among females (APC = 2.1%, 95% CI − 1.3% to 5.6%, *P *=0.218) (Fig. 1a). Age-adjusted incidences (95% CI) were 11.19 (9.71–12.82) per 1,000,000 in the 0- to 1-year-old age group, 5.66 (5.13–6.24) per 1,000,000 in the 2- to 4-year-old age group, and 0.18 (0.14–0.24) per 1,000,000 in the 5- to 18-year-old age group. Incidence among 2- to 4-year-old children increased significantly (APC = 3.6%, 95% CI 1.5% to 5.7%, *P* = 0.003), whereas the increase was not significant among 0- to 1-year-old children (APC = 0.4%, 95% CI − 2.2% to 3.2%, *P* = 0.803) or 5- to 18-year-old children (APC = 0.4%, 95% CI − 3.2% to 4.1%, *P* = 0.782) (Fig. 1b). The incidence among African–Americans also increased significantly (APC = 7.5%, 95% CI 2.5% to 12.8%, *P* = 0.006), which was not the case for Caucasians (APC = 1.8%, 95% CI − 0.2% to 3.8%, *P* = 0.075) and patients of other ethnicities (APC = − 0.1%, 95% CI − 4.4% to 4.4%, *P* = 0.945) (Fig. 1c).

### Survival

**Fig. 2:**
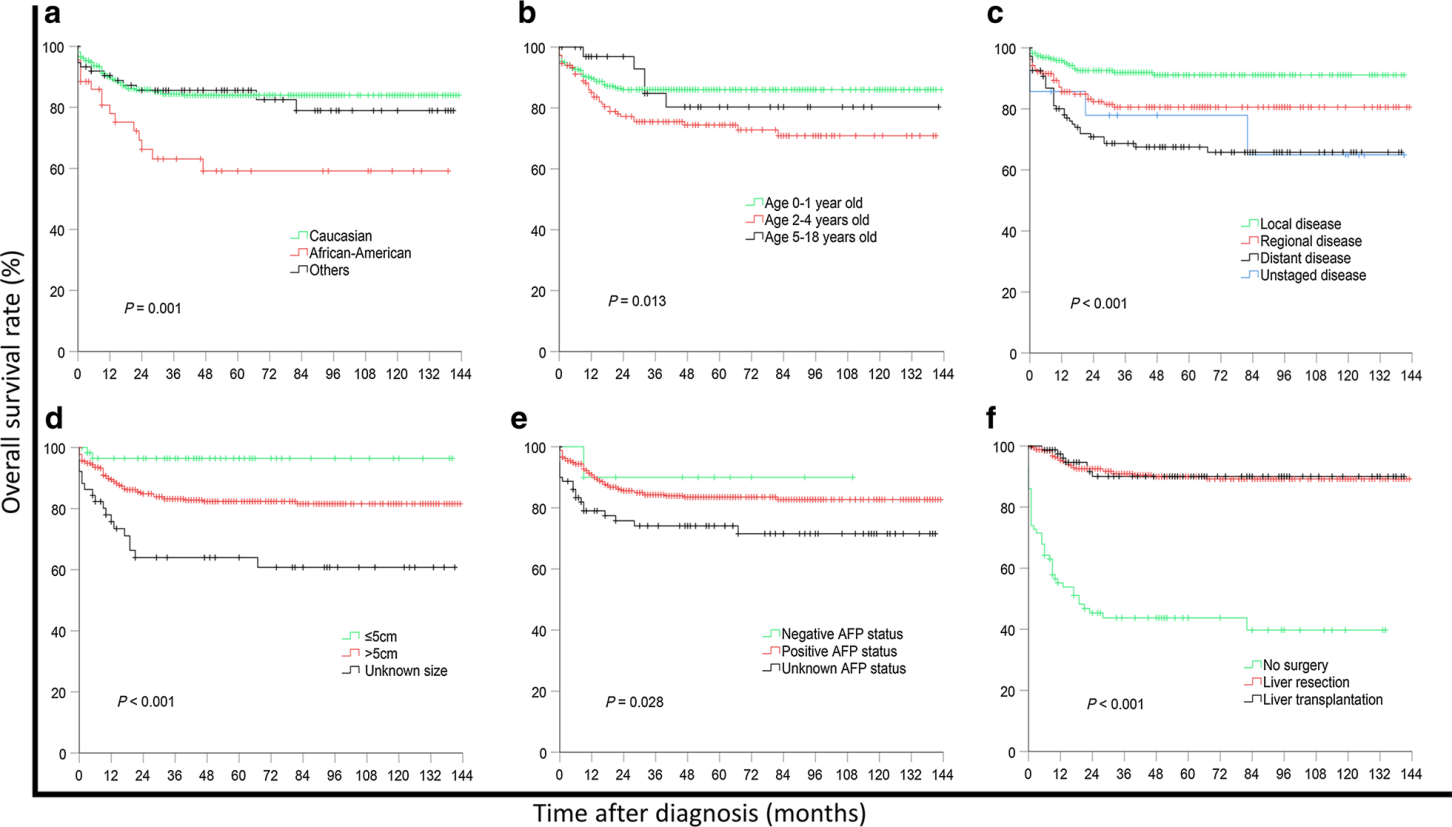
Kaplan–Meier overall survival curves of hepatoblastoma patients stratified by ethnicity (**a**), age at diagnosis (**b**), tumor stage (**c**), tumor size (**d**), AFP status (**e**), and treatment (**f**)

The median follow-up was 45 months (range 0–143 months). Overall, 82 (16.0%) patients died during follow-up. Of these, 46 (56.1%) did not undergo surgery, 29 (35.4%) had liver resection, and 7 (8.5%) had liver transplantation. The 5- and 10-year OS rates were 81.5% and 81.0%, respectively. Risk factors associated with short OS included African–American ethnicity, an age of 2–4 years, distant disease at diagnosis, large tumor size (> 5 cm in diameter), positive AFP status, and not receiving surgery (Table 1, Fig. 2). Type of surgery (liver resection vs. transplantation) did not have an impact on OS rate (*P* = 0.891).

**Fig. 3:**
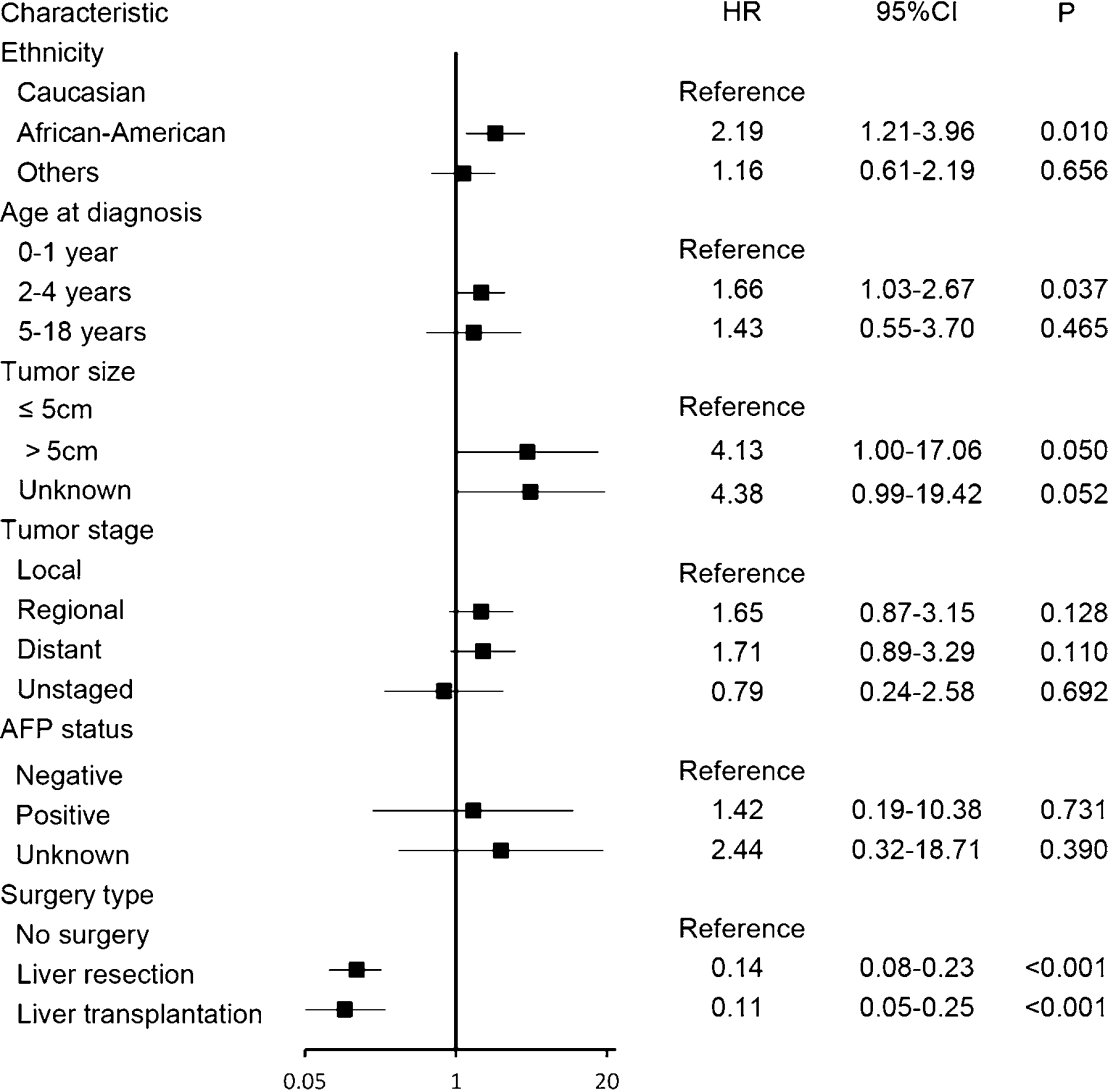
Multivariate analysis of factors of overall survival (OS) of hepatoblastoma patients

Regression analysis using a multivariable Cox proportional hazards model revealed that age of 2–4 years (HR = 1.66, 95% CI 1.03–2.67, *P* = 0.037) and African–American ethnicity (HR = 2.19, 95% CI 1.21–3.96, *P* = 0.010) were independent predictors of shorter OS, whereas liver resection (HR = 0.14, 95% CI 0.08–0.23, *P* < 0.001) and liver transplantation (HR = 0.11, 95% CI 0.05–0.25, *P* < 0.001) were independent predictors of longer OS (Fig. 3). In a multivariate survival analysis that included all variables except for surgery type, tumor stage was found to be an independent factor for OS, but AFP status was still not an independent factor of OS (Table 2).

**Table 2:** Multivariate Cox regression analysis including all variables without surgery type for overall survival of hepatoblastoma patients

Characteristic	HR	95% CI	*P* value
Sex			
Male	Reference		
Female	0.72	0.44–1.18	0.195
Ethnicity			
Caucasian	Reference		
African–American	2.88	1.55–5.36	0.001
Others	1.02	0.54–1.94	0.947
Age at diagnosis (years)			
0–1	Reference		
2–4	1.64	1.03–2.61	0.038
5–18	1.06	0.41–2.71	0.909
Year of diagnosis			
2004–2007	Reference		
2008–2011	0.68	0.40–1.16	0.155
2012–2015	0.84	0.47–1.48	0.540
Tumor size			
≤ 5 cm	Reference		
> 5 cm	3.81	0.92–15.72	0.065
Unknown	6.13	1.39–27.09	0.017
Tumor stage			
Local	Reference		
Regional	2.04	1.10–3.79	0.024
Distant	3.42	1.87–6.26	< 0.001
Unstaged	2.49	0.77–8.07	0.129
AFP status			
Negative	Reference		
Positive	2.19	0.30–16.15	0.442
Unknown	3.01	0.39–23.18	0.291

*HR* hazard ratio, *CI* confidence interval, *AFP* alpha-fetoprotein

### Nomogram for prediction of OS

**Fig. 4:**
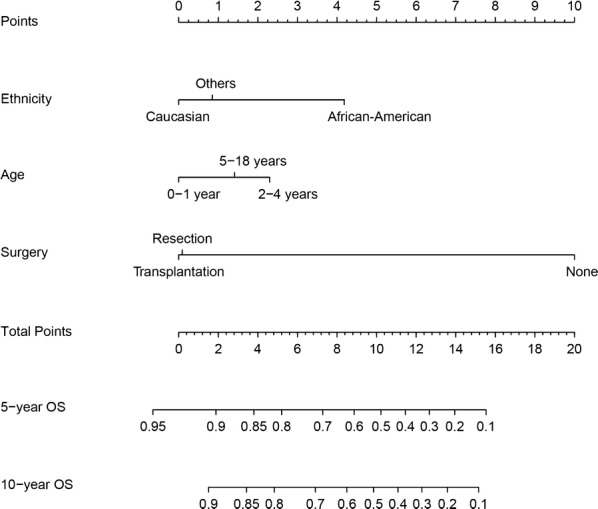
Nomogram for predicting 5- and 10-year overall survival (OS) of hepatoblastoma patients. Minimum and maximum values for OS: Caucasian ethnicity 83.9%–90.1%, African–American ethnicity 58.4%–80.2%, other ethnicities 79.0%–90.1%; 0–1 year old 85.8%–90.0%, 2–4 years old 71.0%–86.0%, 5–18 years old 79.9%–97.0%; no surgery 39.4%–54.6%, liver resection 89.3%–96.2%, liver transplantation 90.1%–97.5%

**Fig. 5:**
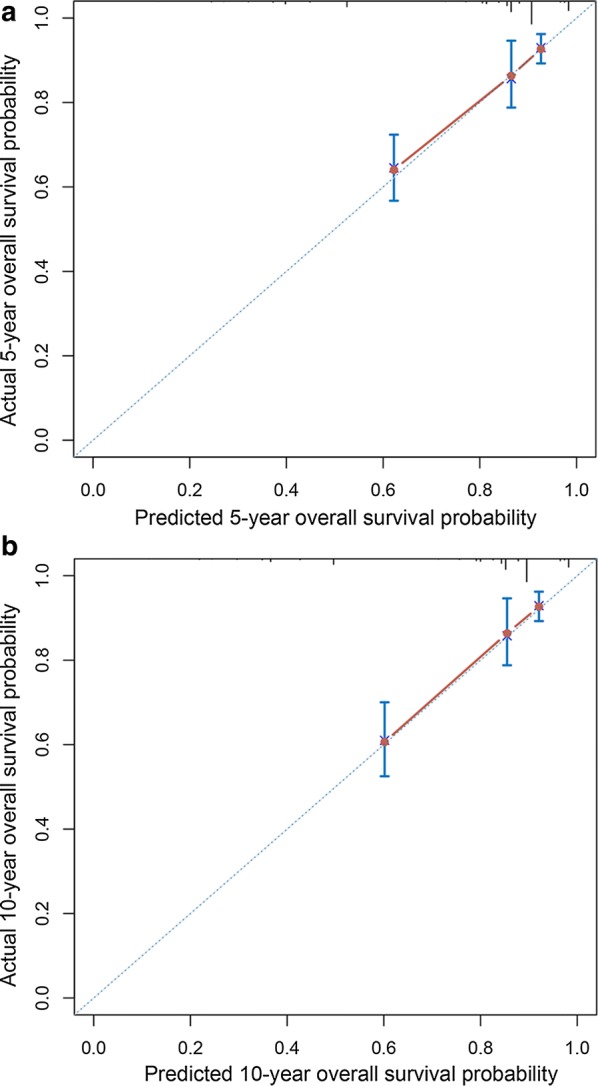
Calibration curves for 5- (**a**) and 10-year overall survival (**b**) of hepatoblastoma patients

We then constructed a nomogram that incorporated all significant prognostic factors identified through multivariate analysis (Fig. 4). The nomogram showed that surgery was the most significant predictor of OS, followed by ethnicity and age. The nomogram also displayed a good C-index of 0.79 (95% CI 0.74–0.84) for predicting OS. The calibration plot for the probability of 5- and 10-year OS showed an optimal agreement between actual observation and the nomogram’s prediction (Fig. 5).

## Discussion

In this population-based study, we assessed temporal trends in pediatric hepatoblastoma incidence and constructed a nomogram to provide an individualized prediction of OS. The current analysis showed a significant annual increase (2.2%) in hepatoblastoma incidence between 2000 and 2015. Although management of this disease has evolved over the past 30 years, there is no model for predicting OS of hepatoblastoma patients. The constructed nomogram was found to be accurate in prognostic prediction for hepatoblastoma patients, with a C-index of 0.79.

Owing to the rarity of hepatoblastoma, single-institution data were insufficient for assessing its incidence trends. Until now, only a few population-based studies have reported hepatoblastoma incidence trends in the past few decades. Darbari et al. [1] reported the incidence of primary liver malignancies among U.S. children aged less than 20 years from 1973 to 1997, using the SEER database. They found that hepatoblastoma incidence had increased, with age-adjusted incidences of 1.09 per 1,000,000 for the whole cohort, 1.22 per 1,000,000 for males, and 0.96 per 1,000,000 for females. However, clinical outcomes and prognostic factors were not reported. In another SEER study of 606 patients diagnosed with hepatoblastoma between 1973 and 2009, Allan et al. [8] found a significant increase in the overall age-adjusted incidence of hepatoblastoma with an APC of 2.18%. However, the reported incidence was not stratified by sex and age. Although outcomes and prognostic factors were reported, the study did not provide a new prognostic model. A recent population-based study analyzing the Taiwan Cancer Registry (TCR) database reported that, from 1995 to 2012, the overall incidence of hepatoblastoma in children increased by 7.4% per year and, specifically, by 6.5% among males [16]. In that study, the incidence of hepatoblastoma was analyzed according to sex and age. However, no outcomes were reported. In the present study, we have provided updated information on hepatoblastoma incidence trends. We found that its incidence increased by 2.2% annually from 2000 to 2015 and, notably, that the incidence increased most significantly among males and in 2- to 4-year-old children.

In the past decades, the management of hepatoblastoma has evolved to include not only surgical treatments such as liver resection and transplantation but also non-surgical treatments such as chemotherapy. Progress in treatment has contributed to prolonged OS in patients with hepatoblastoma. The 10-year OS rate was 81.0% in our analysis, which was better than 61% reported in a SEER-based analysis from 1973 to 2009 [8]. In a single-center retrospective study involving 30 patients younger than 18 years who underwent liver transplantation for hepatoblastoma between 1997 and 2014 at Stanford University School of Medicine, Pham et al. [11] reported 10-year disease-free survival and OS rates of 82% and 84%, respectively. In another SEER study including 318 hepatoblastoma patients undergoing surgery between 1998 and 2009, McAteer et al. [27] found that the overall 5-year disease-specific survival rate was 85.7%, and the rates for patients undergoing resection and transplantation were essentially equivalent (85.6% vs. 86.5%, *P* = 0.66). In the present study, patients who underwent liver resection (*n* = 341) and liver transplantation (*n* = 84) had comparable 10-year OS rates (89.3% vs. 90.1%, *P* = 0.891).

Surgery, ethnicity, and age were identified as independent prognostic factors in the present study. Surgery was a strong predictor of OS. Moreover, the need for liver resection has increased over time: the number of patients receiving liver resection for hepatoblastoma increased by 1.4 folds between the time frames of 2004–2007 and 2012–2015. However, this increase was not observed in patients that received liver transplantation. The number of patients who underwent liver transplantation remained relatively constant between 2004–2007 and 2012–2015. These data emphasize that more children now have the opportunity for a possible cure by surgical resection. With advances in surgical management and chemotherapy, the 5-year OS rate for children with hepatoblastoma has increased to more than 80%. In the current study, we included patients diagnosed between 2004 and 2015 that had been reported in SEER. There is no doubt that these patients mainly benefited from efficient surgical management and the use of novel chemotherapy regimens in the modern era.

It has been reported that age of < 5 years old was a favorable factor for survival [8]. In the present analysis, the 0- to 1-year-old group had the longest OS, whereas the 2- to 4-year-old group had the shortest OS. African–Americans also had shorter OS compared with Caucasians and other ethnicities. This finding was consistent with previous studies [14, 28]. It can be speculated that patients of African–American ethnicity may have had less access to surgical care. Further investigation is necessary to determine if a variation in tumor biology or another etiology is driving this trend. Further surrogate measures, such as poverty/household income, geographic origin of the patient, proximity to surgical centers/tertiary hospitals, or perhaps similarities with the epidemiology of other types of embryonal cancers, can be analyzed in other longitudinal databases with a wider range of epidemiological data as well.

In the present study, distant disease at presentation was not found to be an independent factor for survival. This finding differed from the results of previous studies [8, 14], where it was shown to be a significant prognostic factor. The reason remains unclear, but this may be related to the increased use of chemotherapy, to which hepatoblastoma is highly sensitive [11, 29]. The fact that AFP was not an independent prognostic factor stands out when taking into account existing literature, but this can be attributed to the database effect and also the different use of AFP or the sporadic testing. Further longitudinal studies are needed to determine whether AFP can be used in predicting the prognosis of hepatoblastoma.

In the present study, we constructed a nomogram based on the results of multivariate analysis and included common clinical factors in the model. Our nomogram performed well in predicting OS, and its prediction was supported by the bootstrap-corrected C-index and calibration curve. However, this study contained some limitations. First, information on chemotherapy regimen used was not included, due to incomplete data and biases associated with unstated reasons for receiving or not receiving chemotherapy in the SEER database. Second, although our data were collected from 18 cancer registries of the SEER database, our sample size was still relatively small. The SEER program is a high-quality, population-based cancer registry. Case finding audits of high-volume facilities are routinely performed for data accuracy, completeness, and reporting timeliness. Each SEER registry is given an annual Data Quality Profile, which assesses the extent to which each registry provides data meeting contractual standards. Thus, the SEER registry robustly captures the first course of any cancer-directed surgery. However, given that chemotherapy is routinely used in clinical work, our results represent current treatment outcomes for hepatoblastoma. Third, we could only internally validate the nomogram [30, 31] because of the rarity of hepatoblastoma. Although the nomogram showed good discriminatory ability and the calibration curves were consistent, an external validation of the model is still necessary. Lastly, as a retrospective review, our study is inherently prone to selection bias.

## Conclusions

Our study demonstrated incidence trends of hepatoblastoma in children younger than 18 years and constructed a nomogram to more accurately predict their long-term survival based on a population cohort. The nomogram showed a good predictive performance and may be a useful tool for estimating long-term OS and for better supporting patient counseling.

## Data Availability

SEER Data are available after registration. The authors will provide any additional data if needed.

## Abbreviations

SEER: Surveillance, Epidemiology and End Results
APC: annual percentage change
AFP: α-fetoprotein
OS: overall survival
C-index: concordance index
NCI: National Cancer Institute
SRP: Surveillance Research Program
DCCPS: Division of Cancer Control and Population Sciences
HR: hazard ratio
CI: confidence interval
